# DNA Methyltransferase Inhibition Promotes Th1 Polarization in Human CD4^+^CD25^high^ FOXP3^+^ Regulatory T Cells but Does Not Affect Their Suppressive Capacity

**DOI:** 10.1155/2018/4973964

**Published:** 2018-04-15

**Authors:** Sija Landman, Marjan Cruijsen, Paulo C. M. Urbano, Gerwin Huls, Piet E. J. van Erp, Esther van Rijssen, Irma Joosten, Hans J. P. M. Koenen

**Affiliations:** ^1^Department of Laboratory Medicine-Medical Immunology, Radboud University Medical Center (Radboudumc), Nijmegen, Netherlands; ^2^Department of Hematology, Radboud University Medical Center (Radboudumc), Nijmegen, Netherlands; ^3^Department of Hematology, University Medical Center Groningen, Groningen, Netherlands; ^4^Department of Dermatology, Radboud University Medical Center (Radboudumc), Nijmegen, Netherlands

## Abstract

Regulatory T cells (Treg) can show plasticity whereby FOXP3 expression, the master transcription factor for Treg suppressor function, is lost and proinflammatory cytokines are produced. Optimal FOXP3 expression strongly depends on hypomethylation of the *FOXP3* gene. 5-Azacytidine (Aza) and its derivative 5-aza-2′-deoxycytidine (DAC) are DNA methyltransferase inhibitors (DNMTi) that are therapeutically used in hematological malignancies, which might be an attractive strategy to promote Treg stability. Previous *in vitro* research primarily focused on Treg induction by DAC from naïve conventional CD4^+^ T cells (Tconv). Here, we examined the *in vitro* effect of DAC on the stability and function of FACS-sorted human naturally occurring CD4^+^CD25^high^ FOXP3^+^ Treg. We found that *in vitro* activation of Treg in the presence of DAC led to a significant inhibition of Treg proliferation, but not of Tconv. Although Treg activation in the presence of DAC led to increased IFN*γ* expression and induction of a Thelper-1 phenotype, the Treg maintained their suppressive capacity. DAC also induced a trend towards increased IL-10 expression. *In vivo* studies in patients with hematological malignancies that were treated with 5-azacytidine (Vidaza) supported the *in vitro* findings. In conclusion, despite its potential to increase IFN*γ* expression, DAC does preserve the suppressor phenotype of naturally occurring Treg.

## 1. Introduction

Regulatory T cells (Treg) are important for homeostasis of the immune system [1]. Immune regulation by Treg depends on the stability of these cells [1, 2], which in turn is controlled by stable expression of the transcription factor FOXP3 [3]. In the past, we have shown that Treg reveal plasticity as indicated by loss of FOXP3 expression and gain of proinflammatory cytokine (IL-17a, IFN*γ*) production [4]. Stable FOXP3 expression requires hypomethylation of CpG-rich regions of the *FOXP3* gene, which is known as Treg-specific demethylated region (TSDR) [5–8]. Treg instability and plasticity have been demonstrated in a number of immune-related pathologies and are thought to promote chronic inflammation [9–12]. Demethylating agents, such as the DNA methyltransferase inhibitor (DNMTi) 5-azacytidine (Vidaza, Aza) and its derivative 5-aza 2′-deoxycytidine (decitabine, DAC), are used in the treatment of hematological malignancies and seem an attractive therapeutic strategy to promote Treg stability. Aza and DAC have related mechanisms of action, including depletion of DNMTs and hypomethylation of DNA [13, 14]. Aza/DAC shows immunomodulatory potential *in vitro* and *in vivo* and have been shown to induce demethylation of the FOXP3 gene [15, 16]. Administration of DAC in experimental mouse models of inflammation (lung inflammation [17–19], diabetes [20], colitis [15], multiple sclerosis [21], and GvHD [22]) revealed promising effects on health outcomes. In most of these *in vivo* models, administration of DAC led to an increase in Treg numbers [17, 19, 21, 22] and inhibition of effector cells [21]. In a variety of *in vitro* studies, stimulation of T cells in the presence of DAC led to an increased expression of FOXP3 [15, 23–26] and hypomethylation of the *FOXP3* gene and promoter [15, 22, 24]. Most of these studies focused on the induction of FOXP3 expression in conventional (CD4^+^CD25^−^) T cells [23, 24, 26]. Although DAC treatment induced FOXP3 expression in human CD4^+^CD25^−^ conventional T cells, it is still unclear if DAC induced suppressor potential in these cells [15, 24].

In the clinic, DAC/Aza are used to treat the hematological malignancies myelodysplastic syndrome (MDS), acute myeloid leukemia (AML), and chronic myelomonocytic leukemia (CMML). Overall response rates for Aza and DAC are similar [14]. The working mechanism in these patients is not fully understood, but is supposed to be based on upregulation of antitumor genes [27]. So, on the one hand experiments and clinical data show antitumor properties of these drugs, while other experiments show anti-inflammatory properties.

In MDS, the immune response is altered; previous studies have shown polyclonal/oligoclonal expansion of CD4^+^ and CD8^+^ T cells in both blood and bone marrow [28, 29], changes in the numbers of Treg [30–32], an increase in IL-17A-producing T cells [31], and immune-mediated autologous cytotoxicity against hematopoietic precursor cells [33]. The latter has been proposed to lead to autoimmune myelosuppression and ineffective hematopoiesis [33, 34]. Treg seem to have a role in MDS since in low-risk MDS Treg numbers are reduced, while in high-risk MDS Treg numbers are increased and appear associated with a poor prognosis [35]. Influencing Treg function and stability might be one of the ways in which Aza/DAC sorts its effect in hematological malignancies. However, in patients treated with DAC, conflicting observations were reported regarding the effect of DAC both on CD4^+^ FOXP3^+^ cell numbers and on IFN*γ* and IL-17 production by T cells [23, 26, 36].

Since no data is available on the effect of DAC on FOXP3^+^ Treg stability, suppressive capacity, and function of freshly isolated human naturally occurring CD4^+^CD25^high^ FOXP3^+^ Treg, we here focused on the *in vitro* effect of DAC on the stability and suppressor function of these cells. To put our *in vitro* findings into clinical perspective, we studied FOXP3, Helios, and cytokine expression in CD4^+^ T cells in peripheral blood of patients with hematological malignancies treated with subcutaneous infusion of Vidaza.

## 2. Methods

### 2.1. Patients and Healthy Controls

Peripheral blood (buffy coats) from healthy blood donors was obtained from the blood bank (Sanquin, the Netherlands). Intermediate/high-risk IPSS (International Prognostic Scoring System) patients affected by myeloid dysplastic syndromes(MDS), acute myeloid leukemia (AML), and chronic myelomonocytic leukemia (CMML) (*n* = 14) were treated by s.c. injections of 75 mg/m^2^/day Vidaza on days 1–7 of a 28-day treatment cycle. 10 mL ACDA blood was collected at the start and after 7 days of treatment in the first and fifth treatment cycles. PBMCs were isolated using Lymphoprep (Axis-Shield, Dundee, UK) density isolation. Informed consent was obtained from all patients and healthy blood donors according to the Declaration of Helsinki. The patient study was approved by the METOPP committee (NIPMS-VS-NETH-001, approval number: 469). Patient characteristics are available in Supplemental Table 1.

### 2.2. Cell Isolation and Culture of Cells

Treg and Tconv were isolated from healthy volunteers. CD4^+^ T cells were isolated using RosetteSep™ Human CD4^+^ T cell enrichment Cocktail (StemCell™ Technologies, Vancouver, Canada) according to the manufacturer's instructions. Thereafter, CD4^+^ T cells were labeled with anti-CD25-PE-Cy7 (BC96, eBioscience, San Diego, USA) antibodies to FACS-sort (Aria BD, Franklin Lakes, New Jersey, USA) CD4^+^CD25^−^ (Tconv) and CD4^+^CD25^high^ (Treg). Sorted cells were typically more than 96% pure CD25^high^ cells. Gating strategy is shown in Supplemental Figure 2. Cells were cultured as described previously [37]. In brief, isolated T cells were stimulated with anti-CD3/CD28-mAb-coated beads (T cell expanders, Dynal Biotech, Oslo, Norway), at a bead : cell ratio of 1 : 5 and 100 U recombinant IL-2 (rIL-2, Proleukin, Cetus, Amsterdam, The Netherlands). When indicated, 5-aza-2′-deoxycytidine (DAC, 0.01–100 *μ*M/mL, Sigma-Aldrich, St. Louis, Missouri, USA) and/or IL-1*β* (50 ng/mL, Invitrogen, Waltham, Massachusetts, USA) was added at the start of the cultures.

To study the suppressive capacity of Treg and Tconv that were cultured for 7 days in the presence of DAC, a 5(6)-carboxyfluorescein diacetate *N*-succinimidyl ester- (CFSE-) based coculture suppression assay was performed as described previously [37]. Briefly, decreasing amounts of Treg were cultured with CFSE-labeled autologous T cells. After 4 days, CFSE dilution of the CFSE-labeled T cells was measured using flow cytometry.

### 2.3. Flow Cytometry and Antibodies

Cells were phenotypically analyzed by multicolor flow cytometry (Navios, Beckman Coulter, California, USA). The following cell surface markers were used: anti-CD4-PE-Cy5.5 (MT310, Dako, Santa Clara, California, USA) or anti-CD4-AF700 (RPA-T4, eBioscience), anti-CD25-Pe-Cy7 (BC96, eBioscience) or anti-CD25-APC (2A3, BD), anti-CD45-KO (J33, Beckman Coulter), anti-CD196/CCR6-PE (^∗^11A9, BD), CD183/CXCR3-PerCP5.5 (G025H7, BioLegend, San Diego, California, USA), and CD194/CCR4-PE-Cy7 (BD). To analyze the intracellular expression of FOXP3, Helios, and Ki67, the cells were fixed and permeabilized (Fix-Perm, eBioscience) after surface staining and labeled with anti-FOXP3-eFluor450 (PCH101, eBioscience), Ki67-Alexa-Fluor 488 (B56, BD bioscience), and Helios-Alexa-Fluor 647 (22F6, BioLegend). Intracellular cytokine production was studied after 4 hours of stimulation with PMA (12.5 ng/mL) and ionomycin (500 ng/mL) in the presence of Brefeldin A (5 *μ*g/mL) (all Sigma-Aldrich). After fixation and permeabilization, cells were stained by the following antibodies: anti-IFN*γ*-FITC (4S.B3, BD), anti-IL-17a-PE (EBIO64CAP17, eBioscience), and anti-IL-10-APC (JES3-19F1, BD). Cell viability was analyzed using Fixable Viability Dye eFluor 780 (Cat nr 65-0865, eBioscience). Flow cytometry data were analyzed using Kaluza (version 1.3) software (Beckman Coulter). Cells are gated on lymphocytes based on CD45 staining and forward/side scatter plots. Marker settings were based on isotype controls or unstained cells. Unless mentioned otherwise, graphs present data of day 8.

### 2.4. Real-Time Quantitative Reverse Transcriptase PCR (RT-qPCR)

T cells were cultured as indicated above. At day 4 of the cultures, the cells were harvested and total RNA was extracted by using the RNeasy Plus Micro kit (Qiagen) followed by cDNA synthesis using the SuperScript III First-Strand Synthesis System and Oligo(dT)20 primers (Thermo Fisher Scientific) according to the manufacturer's instruction. TaqMan gene expression assays and primers were purchased from Thermo Fisher Scientific (Supplemental Table 2). The samples were normalized to the C_T_ values of human *HPRT1* (endogenous control). RT-qPCR data were analyzed using the relative quantification app, and 2^−ΔCT^ values were displayed as relative gene expression (Thermo Fisher Scientific Cloud).

### 2.5. Statistics

Statistical analysis was performed using GraphPad Prism version 5.03. The Wilcoxon signed-rank test (2-tailed) was used to test for significance of the findings for *in vitro* studies. For the patient study, paired *t*-tests were used. Differences with a *p* value of <0.05 were considered significant and are indicated with an asterisk (∗). *p* < 0.01 is indicated as ∗∗.

## 3. Results

### 3.1. DAC Promotes Expression of FOXP3 in Treg and Tconv without Altering the Expression of Helios

Previous studies reported the increased expression of FOXP3 in conventional T cells (Tconv) stimulated in the presence of DAC, but these cells did not reveal suppressive capacity [24, 26]. Here, we examined the effect of DAC on the naturally occurring CD4^+^ CD25^high^ FOXP3^+^ Treg population. CD4^+^CD25^high^ cells were isolated by high-purity FACS sorting; >90% of sorted Treg were CD25^+^FOXP3^+^ (Supplemental Figure 1a). Next, dose-response experiments were performed to select the optimal dose of DAC with regard to viability of the cells. FACS-sorted Treg as well as Tconv were stimulated with anti-CD3/CD28 mAb-coated beads, and recombinant human IL-2 (rIL-2) was exogenously added, in the absence or presence of 0.01–100 *μ*M DAC, and cultured for 8 days. The addition of DAC in concentrations up to 1 *μ*M to either stimulated Treg or Tconv did not lead to significant cell death at day 8 of culture (Supplemental Figure 2). Consequently, a dose of 1 *μ*M was used in subsequent experiments. This corresponds with peak concentrations measured in patients treated with decitabine [38].

**Figure 1:**
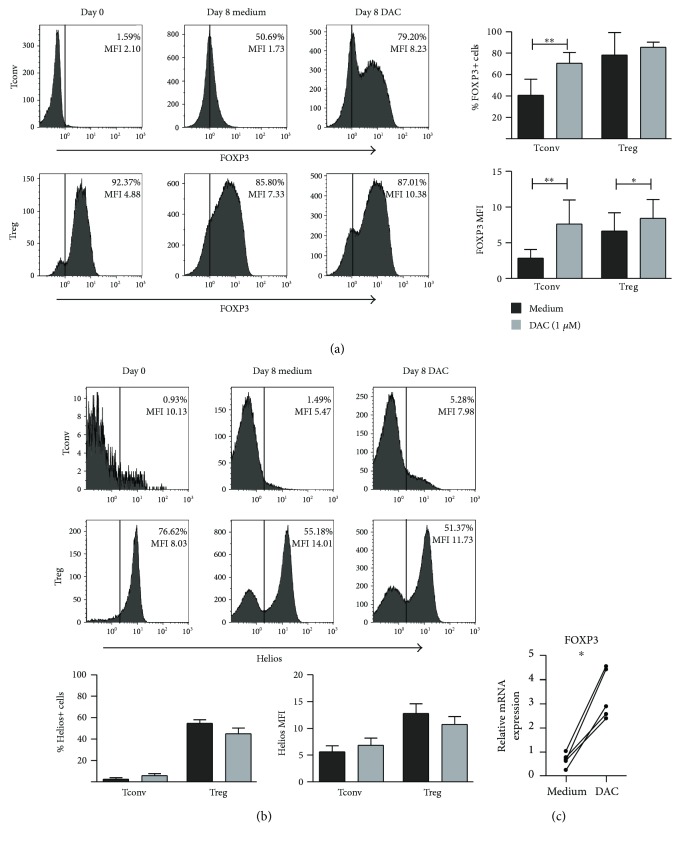
Effect of DAC on FOXP3 and Helios expression of CD4^+^ Treg and Tconv. Flow cytometric analysis of the intracellular expression of (a) FOXP3 and (b) Helios in *α*CD3/*α*CD28^+^ 100U rIL-2-stimulated FACS-sorted Treg and Tconv in the absence or presence of 1 *μ*M of DAC, at day 8 of culture. Cells were gated on the expression of CD45 and CD4. FOXP3 gate settings are based on freshly isolated Tconv and Treg. Representative histograms showing percentage positive cells (%) and median fluorescence intensity (MFI) are presented. Cumulative data presenting the percentage of positive cells and MFI of cells isolated from *n* = 4–7 blood donors are shown. (c) RT-qPCR analysis of FOXP3 expression in isolated CD4^+^ T cells stimulated with *α*CD3/*α*CD28^+^ 100U rIL-2 in the absence or presence of 1 *μ*M of DAC, on day 4 of culture. The samples were normalized to the C_T_ values of human *HPRT1* (endogenous control), and 2^−ΔCT^ values are displayed. *N* = 5 Wilcoxon signed-rank test, 1-tailed, ^∗^ *p* < 0.05, ^∗∗^ *p* < 0.01.

The transcription factors FOXP3 and Helios are important for Treg suppressor function [39]. Here, we assessed the expression of intracellular FOXP3 and Helios in both Treg and Tconv upon anti-CD3/CD28 stimulation in the presence of DAC at day 8 of culture. (Gating strategy is shown in Supplemental Figure 1b.) In Treg, DAC supplementation did not affect the percentage of FOXP3-expressing cells (78.18% ± 21.13 versus DAC 85.38% ± 4.86, NS), but did lead to a significant increase in FOXP3 expression levels (MFI medium 6.63 ± 2.56 versus DAC 8.41 ± 2.67 *p* = 0.0156) (Figure 1(a)). Regarding Helios expression in Treg, addition of DAC led to slightly lower but nonsignificant changes in the percentages of Helios-expressing cells (medium 54.68% ± 3.33 versus DAC 44.90% ± 5.34, NS) and Helios expression levels (MFI medium 12.78 ± 1.80 versus DAC 10.73 ± 1.48, NS) (Figure 1(b)). As shown previously [15, 23, 24], stimulation of FACS-sorted Tconv in the presence of DAC led to a significant upregulation of both FOXP3-expressing cells (medium 40.70% ± 14.95 versus DAC 70.43% ± 10.22, *p* = 0.0078) and FOXP3 expression levels (MFI medium 2.86 ± 1.21 versus DAC 7.62 ± 3.36 *p* = 0.0078) (Figure 1(a)). In these activated Tconv, the expression levels of Helios were low (MFI 2.50 ± 1.34) and were not affected by DAC treatment (5.77 ± 1.92) (Figure 1(b)). Thus, DAC treatment of both Treg and Tconv resulted in an increased FOXP3 expression. The typically high expression levels of Helios in naturally occurring Treg were not affected by DAC; neither did Tconv upregulate Helios expression upon DAC treatment. The increase in FOXP3 expression by DAC was confirmed on the gene expression level using qPCR (Figure 1(c)).

### 3.2. DAC Suppresses Proliferation of Regulatory T Cells but Does Not Alter Their Suppressive Capacity

**Figure 2:**
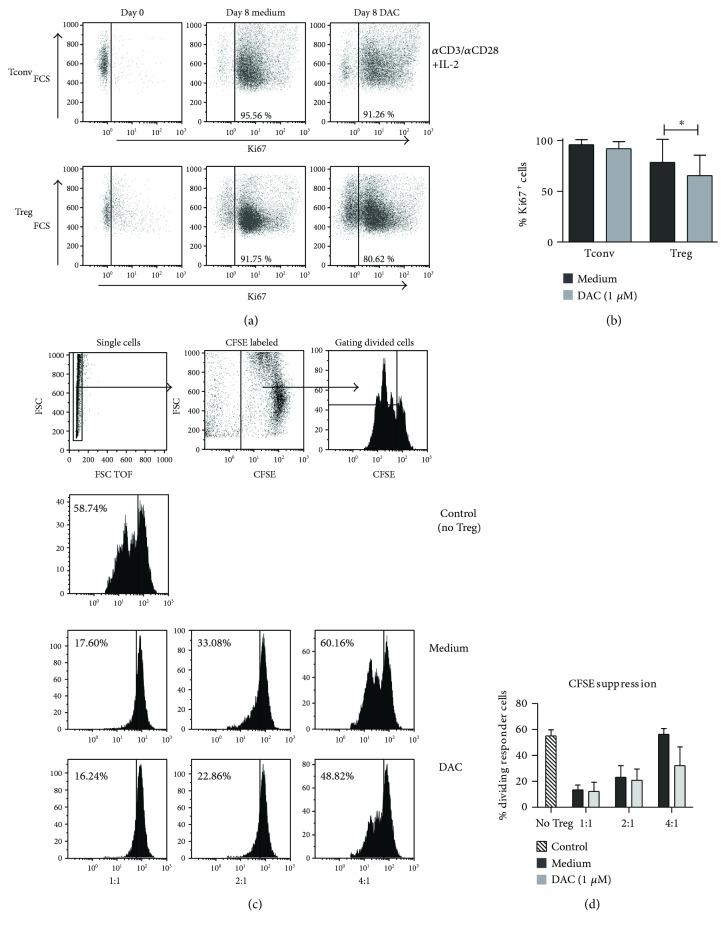
Effect of DAC on proliferation and suppressive capacity of CD4^+^ Treg. (a) Representative dot plots, showing the expression of Ki67 in *α*CD3/*α*CD28^+^ 100U rIL-2 stimulated FACS-sorted Tconv and Treg at day 8 of culture. (b) Cumulative data showing the percentage of Ki67-positive cells in Tconv and Treg stimulated in the presence or absence of 1 *μ*M DAC for 8 days (*n* = 7). (c) Effect of DAC on the suppressive capacity of CD4^+^ Treg. Flow cytometry of a CFSE-based suppression assay of *α*CD3/*α*CD28^+^ 100 U IL-2-stimulated Treg that were cultured for 8 days in the presence or absence of 1 *μ*M DAC. (d) Cumulative data showing the percentage dividing responder cells in the presence of Treg with or without DAC. *N* = 3. Mean ± SD are shown. (b, c) Wilcoxon signed-rank test, 2-tailed, ^∗^ *p* < 0.05.

We then analyzed the effect of DAC on the proliferative capacity of stimulated Treg versus Tconv at day 8 of culture. Addition of 1 *μ*M of DAC significantly inhibited proliferation of Treg as measured by % Ki67 expression (medium 78.37% ± 22.78 versus DAC 65.51% ± 20.06, *p* = 0.0156), but not of Tconv (medium 98.93% ± 4.85 versus DAC 92.07% ± 6.99, *p* = 0.0547) (Figures 2(a) and 2(b)). Cell counts before and after culture confirmed reduced proliferation, which was more prone for Treg as compared to Tconv (data not shown). To determine whether Treg were still suppressive despite inhibited proliferation, a CFSE-based coculture suppression assay was conducted using FACS-sorted Treg that were stimulated and cultured for 8 days in the absence and presence of DAC. DAC-treated Treg kept their suppressive capacity (Figures 2(c) and 2(d)). Although DAC treatment led to an increased FOXP3 expression by Tconv, it did not result in suppressive capacity (not shown), such as that reported previously [24].

### 3.3. Regulatory T Cell Activation in the Presence of DAC Results in Increased IFN*γ* and IL-10 Expression

**Figure 3:**
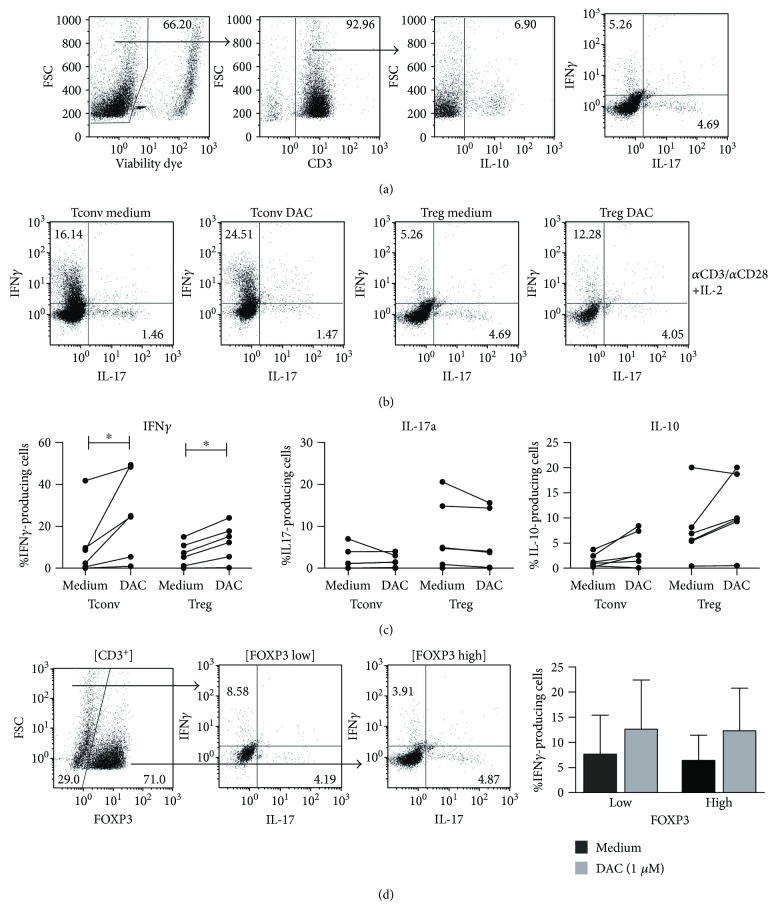
Effect of DAC on cytokine-producing potential of CD4^+^ Treg and Tconv. Flow cytometric analysis of *α*CD3/*α*CD28^+^ 100U rIL-2-stimulated FACS-sorted Treg and Tconv in the absence or presence of 1 *μ*M of DAC, at day 8 of culture. The cytokine-producing potential was analyzed following 4 h stimulation with PMA/ionomycin in the presence of Brefeldin A. (a) Gating strategy. (b) Representative dot plots showing IFN*γ*-, IL-17a-, and IL-10-producing Treg and Tconv that were cultured in the presence or absence of DAC. (c) Cumulative data on IFN*γ*-, IL-17a-, and IL-10-producing Treg and Tconv after culture in the presence of absence of 1 *μ*M DAC. (d) IFN*γ* production in FOXP3^−^ and FOXP3^+^ Treg, representative dot plots, and cumulative data are shown. Mean ± SD are shown. *N* = 6, Wilcoxon signed-rank test, 2-tailed. ^∗^ *p* < 0.05.

The effect of DAC on cytokine expression by T cells is not clear; in some studies, DAC results in upregulation of Th1- and Th17-related cytokines [24], while other studies show downregulation of these cytokines in T cells [21, 23]. To analyze the effect of DAC on the cytokine-producing potential and differentiation of freshly isolated human Treg versus Tconv, these cells were cultured for 8 days with anti-CD3/CD28 mAb-coated beads and rIL-2 in the absence or presence of DAC. Subsequently, expression of intracellular IFN*γ*, IL-17A, and IL-10 was analyzed by flow cytometry. Addition of DAC led to a significantly increased IFN*γ* expression by the stimulated Treg (medium 6.59% ± 5.71 versus DAC 12.45% ± 8.53, *p* = 0.0313), as well as Tconv (medium 10.51% ± 15.83 versus DAC 25.55% ± 20.48, *p* = 0.0313). Also, a trend towards increased IL-10 expression was observed in both Tconv (medium 1.51% ± 1.33 versus DAC 3.70% ± 3.38, *p* = 0.0625 (NS)) and Treg (medium 7.74% versus 6.56 versus DAC 11.37% ± 7.15, *p* = 0.0938 (NS)). The cytokine-producing potential of IL-17A was not influenced by DAC treatment (Figures 3(a)–3(c)). Upon in vitro culture of Treg, a cell population with low FOXP3 expression (FOXP3^low^) and high FOXP3 expression (FOXP3^high^) can be identified [4] (Figure 3(d) **)**. We wondered if cytokine production was associated with the FOXP3 expression levels and subsequently analyzed the cytokine-producing potential in FOXP3^low^ and FOXP3^high^ CD4^+^ cells. No differences were found in cytokine expression between FOXP3^low^ and FOXP3^high^ cells (Figure 3(d)).

### 3.4. Regulatory T Cells Polarize towards a Th1-like Phenotype upon DAC Treatment

**Figure 4:**
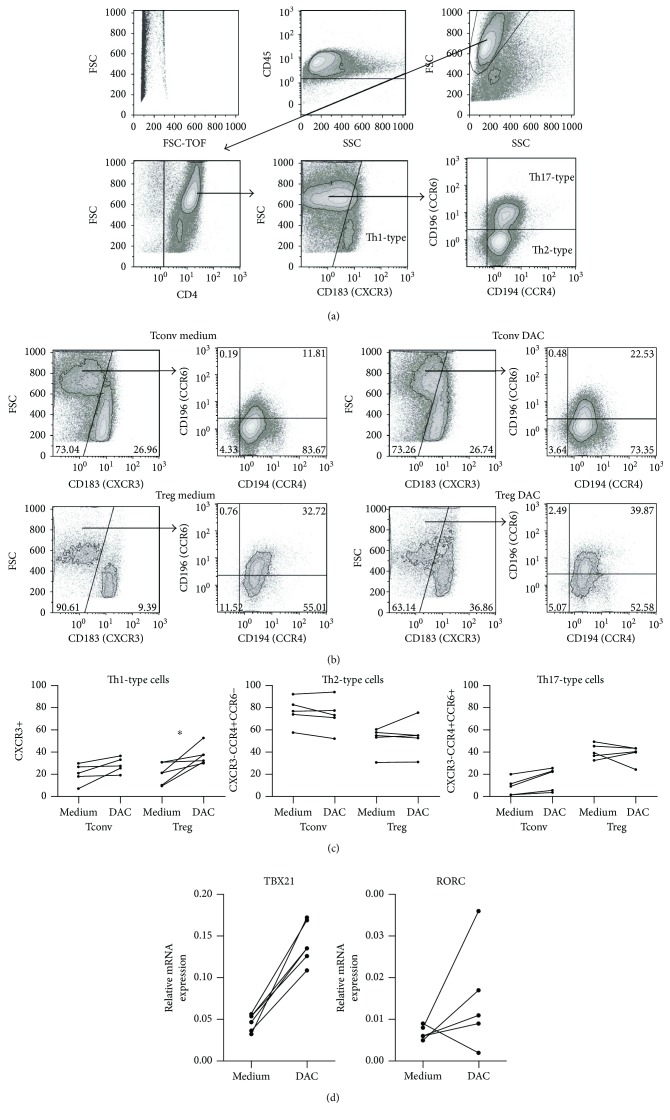
Effect of DAC on chemokine receptor expression of CD4^+^ Treg and Tconv. Flow cytometry of *α*CD3/*α*CD28^+^ 100 U IL-2-stimulated FACS-sorted Treg and Tconv on day 8 of culture. (a) Gating strategy and definition of Th1/Th2/Th17(like) Tconv and Treg. (b) Representative dot plots of Th1/Th2/T17(like) cells of Tconv and Treg that were cultured in the presence or absence of DAC. (c) CXCR3^+^ cells (Th1), CXCR3^−^CCR4^+^CCR6^−^ cells (Th2), and CXCR3^−^CCR4^+^CCR6^+^ cells (Th17) *N* = 4–5, Wilcoxon signed-rank test, 2-tailed. ^∗^ *p* < 0.05. (d) RT-qPCR analysis of TBX21 (Tbet) and RORC expression in isolated CD4^+^ cells stimulated with *α*CD3/*α*CD28^+^ 100U rIL-2 in the absence or presence of 1 *μ*M of DAC, on day 4 of culture. The samples were normalized to the C_T_ values of human *HPRT1* (endogenous control), and 2^−ΔCT^ values are displayed. *N* = 5 Wilcoxon signed-rank test, 2-tailed, ^∗^ *p* < 0.05.

Analysis of the expression levels of the chemokine receptors CXCR3, CCR6, and CCR4 enables further characterization of peripheral blood Thelper-like subsets [35–37]. Th1-like cells are contained within the CXCR3^+^ cell population, while Th17-like and Th2-like cells are contained within CXCR3^−^CCR4^+^CCR6^+^ and CXCR3^−^CCR4^+^CCR6^−^ cell populations, respectively. Following DAC treatment of stimulated Treg and Tconv, an increase in CXCR3-expressing cells was observed in both Treg (medium 20.72% ± 9.357 versus DAC 36.89% ± 8.518, *p* = 0.0313) and Tconv (medium 20.69% ± 8.729 versus DAC 28.44% ± 6.712, *p* = 0.0625 (NS)) (Figure 4). To confirm strong Th1 polarization by DAC in CD4^+^ T cells, we analyzed the expression of the Th1 master transcription factor Tbet by RT-qPCR after stimulation of isolated CD4^+^ T cells in the absence or presence of DAC. Supplementation of DAC led to a significant increase in TBX21 (encoding Tbet) mRNA expression (medium 0.045 ± 0.0107) versus DAC 0.1422 ± 0.0275, *p* = 0.0313), while mRNA expression of the prototypic Th17 transcription factor RORC was not influenced by DAC. Together with the above observed increase in IFN*γ* expression, this suggests that DAC favors differentiation towards a Th1-like phenotype. DAC did not significantly influence Th2- and Th17-associated marker expression.

To confirm strong Th1 polarization by DAC in CD4^+^ T cells, we analyzed the expression of the Th1 master transcription factor Tbet by RT-qPCR after stimulation of isolated CD4^+^ T cells in the absence or presence of DAC. Supplementation of DAC led to a significant increase in TBX21 (encoding Tbet) mRNA expression (medium 0.045 ± 0.0107) versus DAC 0.1422 ± 0.0275, *p* = 0.0313), while mRNA expression of the prototypic Th17 transcription factor RORC was not influenced by DAC. Together with the above observed increase in IFN*γ* expression, this suggests that DAC favors differentiation towards a Th1-like phenotype.

### 3.5. The Effect of DAC on Treg under Proinflammatory Conditions

**Figure 5:**
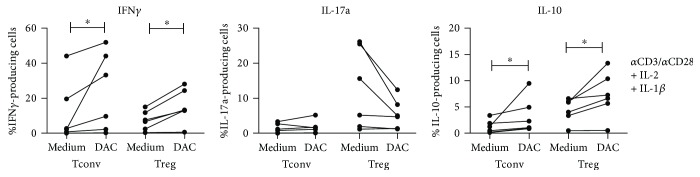
Effect of DAC on cytokine-producing potential of CD4^+^ Treg under proinflammatory conditions. Flow cytometric analysis of *α*CD3/*α*CD28^+^ 100U IL-2 + 50 ng/mL IL-1*β*-stimulated FACS-sorted Treg and Tconv on day 8 of culture. The cytokine-producing potential was analyzed following 4 h stimulation with PMA/ionomycin in the presence of Brefeldin A. Cumulative data on IFN*γ*-, IL-17a-, and IL-10-producing Treg and Tconv that were cultured in the presence or absence of 1 *μ*M DAC. Mean ± SD are shown. *N* = 6, Wilcoxon signed-rank test, 2-tailed. ^∗^ *p* < 0.05.

Previously, we showed that IL-17A production by Treg was increased under proinflammatory conditions, which could be prohibited by treatment with epigenetic modifiers, like trichostatin A [4]. Here, we examined whether DAC had similar effects. To mimic a proinflammatory condition, rIL-1*β* was added on day 0 to cultures of CD3/CD28 bead and rIL-2-stimulated Treg (Tconv were included for comparison) and IL-17A, IFN*γ*, and IL-10 expression was analyzed on day 8 by flow cytometry. DAC led to a significant increase of the IFN*γ*-producing capacity in both Treg (medium 7.32% ± 5.53 versus DAC 15.40% ± 9.76, *p* = 0.0313) and Tconv (medium 11.58% ± 17.56 versus DAC 23.57% ± 22.41, *p* = 0.0313). Also, a significant increase in IL-10 was observed under these conditions in both Treg (medium 4.40% ± 2.30 versus DAC 7.30% ± 4.36) and Tconv (medium 1.23% ± 1.26 versus DAC 3.27% ± 3.42). The effect of DAC on IL-17A production by Treg was only apparent in three high producers of IL-17A (>10% IL-17A-producing cells following IL-1*β* stimulation); here, DAC treatment led to inhibition of IL-17A expression. In the low IL-17A producers as well as in Tconv, no effect was seen (Figure 5).

### 3.6. *In Vivo* 5-Azacitidine (Vidaza) Has the Potential to Promote IFN*γ* Expression in Patients with Hematological Malignancies

DAC-based demethylating agents are used to treat hematological conditions such as MDS, AML, and CMML. Previous studies on *in vivo* treatment with AZA/DAC in these patients reported conflicting observations with respect to the effect of AZA/DAC on Treg numbers and cytokine production [23, 26, 36]. To support our *in vitro* findings, we performed an ex vivo analysis of peripheral blood T cells in 14 patients with MDS, AML, and CMML that were treated with Vidaza, for 7 consecutive days, every 28 days. Peripheral blood samples were analyzed for intracellular FOXP3 and IFN*γ*, IL-17a, and IL-10 expression by CD3^+^CD8^−^ cells (“CD4+ cells”) and CD3^+^CD8^−^FOXP3^+^ T cells using flow cytometry.

**Figure 6:**
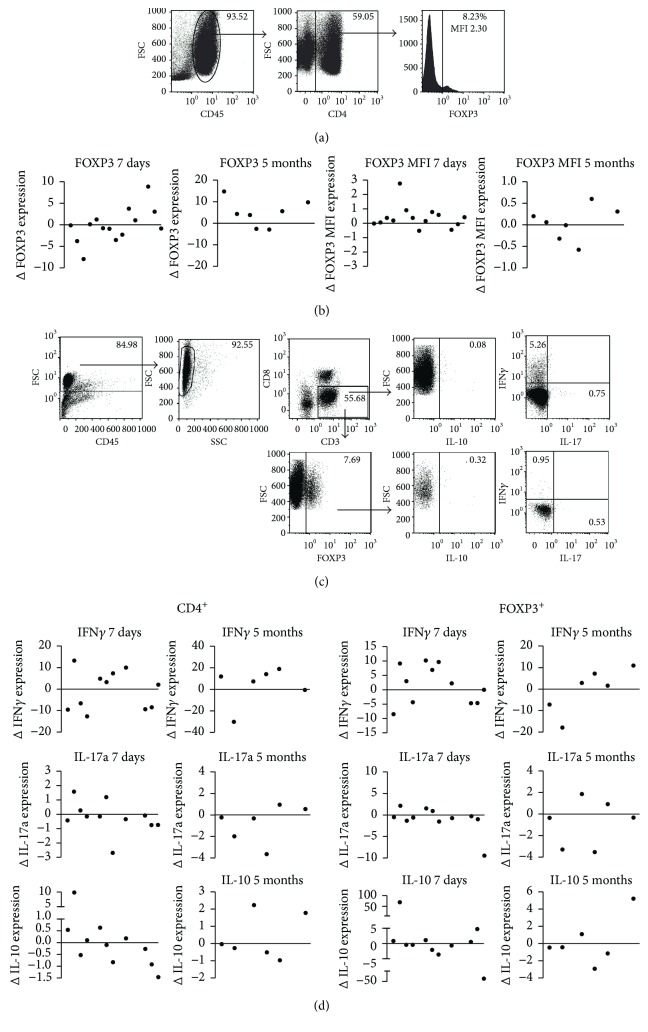
*In vivo e*ffect of Vidaza treatment on FOXP3 expression and cytokine expression by CD4 T cells. Flow cytometry of isolated PBMCs obtained from Vidaza-treated patients with hematological malignancies. (a) Gating strategy used for FOXP3 analyses. (b) Difference (Δ) in percentage FOXP3^+^ cells and MFI after 7 days (short term) (*N* = 11) and after 5 cycles (long term) (*N* = 7) of Vidaza. (c) Gating strategy used for cytokine analysis in CD4^+^ and CD4^+^FOXP3^+^ T cells after 4 h PMA/ionomycin stimulation in the presence of Brefeldin A. (d) Difference (Δ) in percentage of IFN*γ*, IL-17A, and IL-10 in CD4^+^ T cells and CD4^+^FOXP3^+^ Treg after 7 days and 5 cycles of Vidaza. Paired *t*-test, no significant differences.

Vidaza treatment resulted in an increased FOXP3 expression level in 10 out of 14 patients after 7 days of treatment. Seven of these patients were still participating in the study after 5 cycles of Vidaza treatment; 4 out of 7 patients showed an increased FOXP3 expression level (NS) (Figures 6(a) and 6(b)). After 7 days of treatment, IFN*γ* expression was increased in 6 out of 11 patients in both CD4^+^ cells and CD4^+^FOXP3^+^ cells; however, this was not significant. After 5 cycles, 4 of 6 patients show an increase in IFN*γ* expression in both CD4^+^ and CD4^+^FOXP3^+^ cells (NS). IL-10- and IL-17a-producing capacity is decreased in the majority of patients. IL-10 was decreased in 6/11 patients after 7 days and in 4/6 after 5 cycles, both in CD4^+^ and CD4^+^FOXP3^+^. IL-17a was decreased in 8/11 patients after 7 days and in 4/6 patients after 5 months (NS) (Figures 6(c) and 6(d)).

The increased expression levels of FOXP3 (MFI) and IFN*γ* observed in the majority of patients resembled the *in vitro* data. The differences in FOXP3 expression and cytokine production were not correlated with the clinical outcomes such as survival, hematological improvement, or transfusion response.

## 4. Discussion

Promoting Treg numbers and improving their stability appear an attractive approach to prevent inflammation in a variety of immunopathogenic processes [40–43] and is considered a means to prevent myelosuppression and ineffective hematopoiesis in patients with hematological malignancies [33, 34]. In these patients, the immune system is changed, as for example indicated by clonal expansion of CD4^+^ and CD8^+^ T cells and variable numbers of Treg depending on disease severity [30–32]. The DNMT inhibitors Aza and DAC, which seemingly have the potential to promote FOXP3 expression [15, 17, 21, 22], are therapeutically used in MDS, AML, and CMML patients to reduce uncontrolled myelodysplasia and increased survival of the patients [34, 44]. This DAC treatment also influences the immune system of the treated patients as indicated by changes in Thelper cells, regulatory T cells, and the CD4/CD8 T cell composition [23, 26, 36]. Variation in Treg numbers in peripheral blood of MDS patients has been reported [30–32], and expansion of Treg in high-risk MDS correlates with a poor prognosis [35], suggesting that Treg play a role in this disease. It is not known if DAC affects human naturally occurring FOXP3^+^ Treg in their function.

In the presented work, we demonstrate that supplementation of DAC to *in vitro* cultures of naturally occurring Treg induces increased FOXP3 expression levels without influencing Helios expression levels. DAC inhibits Treg proliferation; yet, DAC-treated Treg maintain their suppressor capacity. However, even though DAC-treated Treg cells are still suppressive, DAC-treated Treg increase their IFN*γ*-producing potential. Under inflammatory conditions, not only did both Tconv and Treg upregulate IFN*γ* but also IL-10 was significantly increased upon DAC supplementation. Increased FOXP3 expression levels and IFN*γ* production were also observed in patients treated with Vidaza.

It has previously been shown that Treg numbers are significantly increased in high-risk MDS, whereas in low-risk MDS IL-17-producing CD4^+^ T cells were increased, which suggests an association between the number of Treg and Th17 and disease severity [31, 35]. Expansion of regulatory T cells occurs in high-risk MDS and correlates with a poor prognosis [35]. We here show that although DAC supplementation supports Treg suppressor functions, it inhibits Treg proliferation, which might contribute to the mechanism of action of DAC in high-risk MDS. However, in various inflammatory mouse models for diabetes, multiple sclerosis, lung inflammation, and GVHD, Treg numbers were increased. Increased Treg numbers were designated as the mechanism to prevent immune pathology [17, 19–22, 45]. In one study, it was shown that Treg from DAC-treated mice revealed increased *in vitro* suppressive potential [19], while other studies failed to demonstrate the increased suppressive capacity by Treg from DAC-treated mice [7, 15]. Conflicting observations were reported regarding the effect of DAC on Treg numbers *in vivo in* humans; Costantini et al. [26] and Bontkes et al. [36] reported that 9 months (68 patients) or 3 months (9 patients) of DAC treatment of MDS patients did not result in an increase of FOXP3-expressing cells, while Stübig et al. [23] and Schroeder et al. [25] found increased Treg numbers in AML, CMML, and MDS patients.

In our patient study, we studied 14 patients. In these patients, the majority, 10 out of 14, showed an increase in FOXP3 expression after 7 days of treatment, but after 6 months this effect was no longer evident. From our data, it seems that the effect exerted by DAC on Treg in vivo is mainly a short-term effect and not a persisting long-term effect. This is supported by the studies of Stübig et al. [23] and Schroeder et al. [25], which reveal that measuring relatively early after DAC/Aza treatment results in increased FOXP3 expression levels, while the studies of Costantini et al. [26] and Bontkes et al. [36], which measure after a prolonged treatment, did not reveal differences in FOXP3 expression levels. This might also explain differences in the outcome between the different studies. It should however be noted that we cannot rule out that the short-term increase in the prevalence of FOXP3^+^ T cells is the result of transient FOXP3 expression by activated CD4^+^ T cells. Given the above information, it is not likely that Treg numbers and/or stability is crucial for the long-term successful DAC effects in hematological malignancies.

Inhibition of proliferation of Tconv is one of the mechanisms by which Treg exert their immune suppressive effect. We here demonstrate that Treg, which were cultured in the presence of DAC, maintained their suppressive potential, while culture of Tconv in the presence of DAC does not confer suppressor potential despite the fact that these cells showed an increased FOXP3 expression following DAC treatment, such as that also reported by Kehrmann et al. [24]. This is in contrast to the observations by Lal et al. [15], who showed that DAC-treated Tconv did become suppressive. Notably, at present there is much debate on the association of FOXP3 expression in Tconv and their immune suppressive potential [46].

*In vitro*, we observed an increased IFN*γ* expression upon DAC and Vidaza treatment, suggesting that reduced methylation promotes the IFN*γ*-producing capacity of T cells. After 5 months of treatment, this was also seen ex vivo in patients with hematological malignancies, in 4 out of 6 patients. This is in line with findings in mice, showing that deletion of the Dnmt1 gene that promotes reduced methylation led to an upregulation of cytokine-encoding genes including IFN*γ* in mouse T cells [47, 48]. Vice versa, in mice it was shown that increased methylation inhibits IFN*γ* transcription in T cells [49]. Regarding the effect of DAC on IFN*γ* expression by human T cells, it was observed that DAC led to an increase in IFN*γ*-expressing CD4^+^ T cells *in vitro* in T cells isolated from MDS patients but not in cells isolated from healthy donors [26]. In myelodysplasia patients treated with DAC, IFN*γ* expression by CD4^+^ T cells was not promoted [26, 36]. In two *in vitro* studies, contradictory observations were reported with respect to the effect of DAC on IFN*γ* expression in healthy donors [23, 36]. Our study demonstrates that DAC promotes IFN*γ* expression upon *in vitro* activation of Tconv obtained from healthy donors. Moreover, in our study, we demonstrate for the first time that this is also the case for Treg. Despite the increased IFN*γ* expression following *in vitro* stimulation of naturally occurring Treg in the presence of DAC, the Treg maintained their suppressor potential and also still produce IL-10. It has been demonstrated before that expression of proinflammatory cytokines by Treg does not hamper their suppressor capacity *in vivo* [50]. In fact, recently it has been demonstrated that Th1-like Treg are crucial for the protection of diabetes in a mouse model [51]. Th1-like Treg were also observed in MS patients; next to IFN*γ*, an increase in IL-10 by these cells was observed [50].

Based on chemokine expression profiles, different Thelper populations have been described in Treg and Tconv [52]. We here demonstrate that next to an increase in IFN*γ* expression, also the percentages of CXCR3^+^ Thelper 1-like Treg and Tconv were increased following DAC supplementation in vitro. Additional to the increase in IFN*γ* following DAC supplementation, we observed a trend towards increased IL-10 expression by Treg and Tconv. It has been previously postulated that IL-10 is frequently coproduced with IFN*γ* in Th1-like Treg populations [52], while conventional Th1 cells rarely produce IL-10 [52]. However, under proinflammatory stimulatory conditions (i.e., anti-CD3/CD28 beads, IL-2, and IL-1*β*), we observed that DAC supplementation led to a significant increase in IL-10 expression by CD4^+^ Tconv and Treg. The increase in IL-10 was not observed in patients, which might be due to the limited numbers of Treg in peripheral blood and the limited IL-10 expression of freshly isolated cells.

Whether AZA/DAC has a proinflammatory or anti-inflammatory outcome seems to depend on the nature of the disease, disease state, and dosing of the drug. More research in this field is necessary to find the optimal treatment regimen for cancer patients and to further explore possibilities of using DNMT is in inflammatory diseases.

In conclusion, the DNMT inhibitor DAC promotes Th1 polarization of Treg *in vitro. In vivo*, a similar increase in IFN*γ* was observed. Despite the upregulation of IFN*γ* and the inhibition of proliferation, Treg do maintain their suppressive capacity. This might be due to the increased production of IL-10. The outcome of AZA/DAC treatment seems to be dependent on a delicate balance between proinflammatory and anti-inflammatory processes and cytokines produced.

## Supplementary Materials

Supplementary Materials Supplemental Figure 1: FACS settings and gating strategy of CD4^+^ Treg and Tconv. (A) Gating strategy used for FACS and rerun of CD4^+^CD25^high^-sorted cells; %CD4^+^CD25^+^FOXP3^+^ after sorting (day 0). (B) Gating strategy. Sorted Tconv (CD25^−^) and Treg (CD25^high^) cells are gated on CD45^+^ and CD4^+^ to analyze the expession of FOXP3 and CD25. Supplemental Figure 2: effect of DAC on viability of CD4^+^ Treg and Tconv. Flow cytometric analysis of *α*CD3/*α*CD28^+^ 100U rIL-2 stimulated CD4^+^CD25^high^ (Treg) and CD4^+^CD25^−^ (Tconv) FACS-sorted T cells at day 8 of culture. Mean ± SD is shown. Dose-response curve showing the effect of increasing concentrations of DAC (*x*-axis) on the viability of Treg and Tconv as determined by labelling with the Fixable Viability Dye eFluor 780 (*y*-axis) (*n* = 2). Supplemental Table 1: patient characteristics.
