# Supplementary material for: DNA Methyltransferase Inhibition Promotes Th1 Polarization in Human CD4+CD25high FOXP3+ Regulatory T Cells but Does Not Affect Their Suppressive Capacity
Source: J Immunol Res. 2018 Apr 15;2018:4973964. doi: 10.1155/2018/4973964 (PMC5924998; doi:10.1155/2018/4973964)
Supplement: Supplementary Materials — Supplemental Figure 1: FACS settings and gating strategy of CD4+ Treg and Tconv. (A) Gating strategy used for FACS and rerun of CD4+CD25high-sorted cells; %CD4+CD25+FOXP3+ after sorting (day 0). (B) Gating strategy. Sorted Tconv (CD25−) and Treg (CD25high) cells are gated on CD45+ and CD4+ to analyze the expession of FOXP3 and CD25. Supplemental Figure 2: effect of DAC on viability of CD4+ Treg and Tconv. Flow cytometric analysis of αCD3/αCD28+ 100U rIL-2 stimulated CD4+CD25high (Treg) and CD4+CD25− (Tconv) FACS-sorted T cells at day 8 of culture. Mean ± SD is shown. Dose-response curve showing the effect of increasing concentrations of DAC (x-axis) on the viability of Treg and Tconv as determined by labelling with the Fixable Viability Dye eFluor 780 (y-axis) (n = 2). Supplemental Table 1: patient characteristics. [file 4973964.f1.pdf]

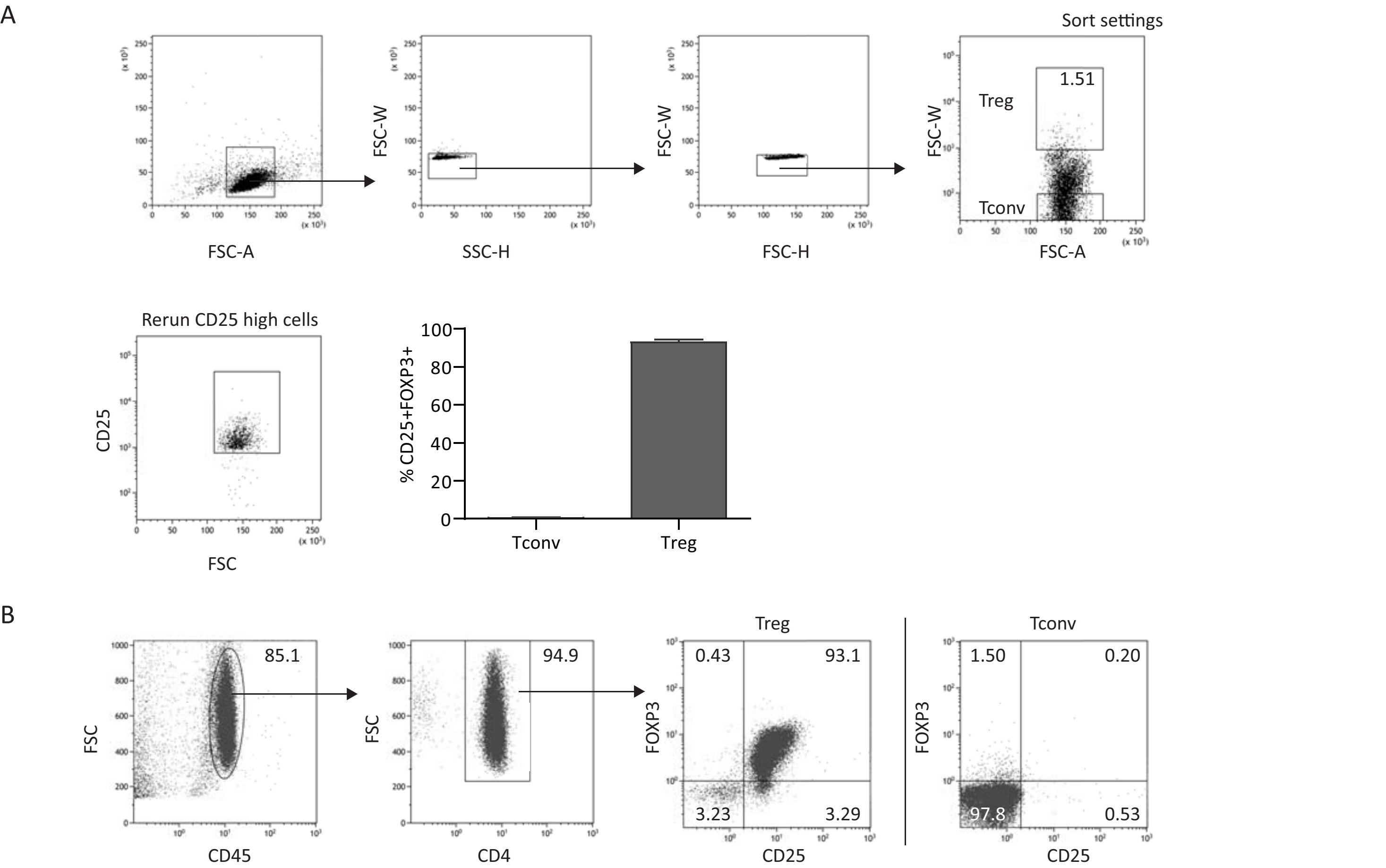
A
Sort settings
Treg
1.51
Tconv
FSC-W
FSC-A
SSC-H
FSC-H
Rerun CD25 high cells
CD25
FSC
% CD25+FOXP3+
100
80
60
40
20
0
Tconv
Treg
B
85.1
94.9
FSC
CD45
CD4
Treg
0.43
93.1
3.23
3.29
Tconv
1.50
0.20
97.8
0.53
FOXP3
CD25

Supplemental figure 2

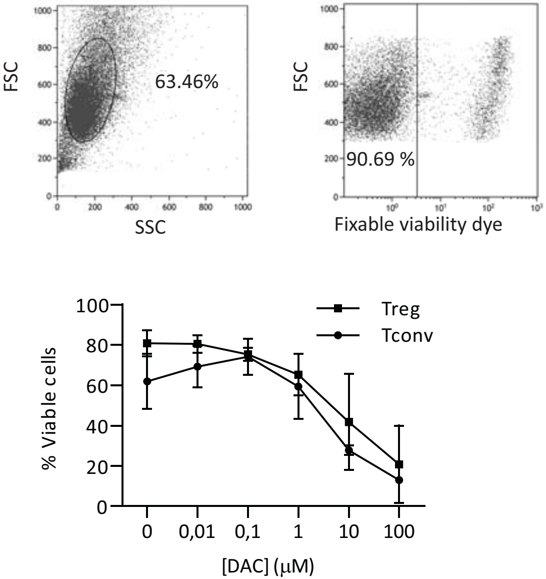

pt #	Age	Diagnosis	Cycles	Reason of discontinuation
1	71	CMML-2	2	Died of hart failure
2	79	AML multilineage dysplasia	>5	Ongoing
3	68	CMML-2	4	Stop due to toxicity
4	70	AML multilineage dysplasia	>5	Ongoing
5**	70	MDS-t	>5	Ongoing
6	76	AML	>5	Ongoing
7	70	AML	3	Ongoing, quit study
8	53	CMML-1	4	Ongoing, quit study
9	78	MDS-RAEB2	3	Stop due to toxicity
10*	77	CMML-2	>5	Ongoing
11	97	MDS-RAEB2	5	Palliative
12	76	MDS-RAEB-1	>5	Ongoing
13	79	AML M2	>5	Ongoing
14	82	MDS-RAEB-1	>5	Ongoing
15**	71	AML	2	Changed to other study

Patients with >5 cycles are included in the 5 cycles analysis

* Pt 10 was excluded from the analysis because no blood was drawn in first cycle

** No cytokines measured before start of treatment
